# Comprehensive benchmarking of software for mapping whole genome bisulfite data: from read alignment to DNA methylation analysis

**DOI:** 10.1093/bib/bbab021

**Published:** 2021-02-23

**Authors:** Adam Nunn, Christian Otto, Peter F Stadler, David Langenberger

**Affiliations:** ecSeq Bioinformatics GmbH, Sternwartenstraße 29, 04103, Saxony, Germany; Institut für Informatik, Universität Leipzig, Härtelstraße 16-18, 04107, Saxony, Germany; ecSeq Bioinformatics GmbH, Sternwartenstraße 29, 04103, Saxony, Germany; Institut für Informatik, Universität Leipzig, Härtelstraße 16-18, 04107, Saxony, Germany; ecSeq Bioinformatics GmbH, Leipzig, Germany

**Keywords:** whole genome bisulfite sequencing, DNA methylation, benchmark, WGBS mapping software, non-model plants, epigenetics

## Abstract

Whole genome bisulfite sequencing is currently at the forefront of epigenetic analysis, facilitating the nucleotide-level resolution of 5-methylcytosine (5mC) on a genome-wide scale. Specialized software have been developed to accommodate the unique difficulties in aligning such sequencing reads to a given reference, building on the knowledge acquired from model organisms such as human, or *Arabidopsis thaliana*. As the field of epigenetics expands its purview to non-model plant species, new challenges arise which bring into question the suitability of previously established tools. Herein, nine short-read aligners are evaluated: *Bismark*, *BS-Seeker2*, *BSMAP*, *BWA-meth*, *ERNE-BS5*, *GEM3*, *GSNAP*, *Last* and *segemehl*. Precision-recall of simulated alignments, in comparison to real sequencing data obtained from three natural accessions, reveals on-balance that *BWA-meth* and *BSMAP* are able to make the best use of the data during mapping. The influence of difficult-to-map regions, characterized by deviations in sequencing depth over repeat annotations, is evaluated in terms of the mean absolute deviation of the resulting methylation calls in comparison to a realistic methylome. Downstream methylation analysis is responsive to the handling of multi-mapping reads relative to mapping quality (MAPQ), and potentially susceptible to bias arising from the increased sequence complexity of densely methylated reads.

## 1 Introduction

Over the three decades following the conception of bisulfite sequencing by Frommer *et al.* [1] it has become the foundation of many investigations linking DNA methylation with epigenetics at nucleotide-level resolution. DNA can undergo a number of base modifications with nearly 40 having been verified in the DNAmod database [2] as of the date of publication. Cytosine methylation is among the most abundant of these in eukaryotes, involving the addition of a methyl group (CH$_3$) to the 5th carbon position of the cytosine ring to form 5-methylcytosine (5mC). In model plants and crops, 5mC has been associated with changes in gene expression [3–5], chromosome interactions [6, 7] and genome stability through the repression of transposable elements [8, 9]. The role of 5mC in epigenetics is well studied in model organisms, but with falling sequencing costs and advances in modern sequencing technology there is incentive now to extend this research to non-model species.

DNA samples are treated with sodium bisulfite during library preparation [10], which facilitates the deamination of unmethylated cytosines to uracil while methylated bases remain unaffected. During the first round of replication uracil pairs with adenosine rather than guanosine, which in-turn pairs with thymine in the amplified polymerase chain reaction (PCR) product of the original sequence. Unlike standard sequencing, the library after PCR amplification contains four distinct read-types: the forward and reverse complements of the converted sequence on the Watson(+) strand, and also the forward and reverse complements of the converted sequence on the original complementary Crick(-) strand. After mapping, the converted bases can then be cross-referenced with the known genome to distinguish between converted cytosines and true thymines. Unconverted cytosine bases indicate the presence of 5mC.

The alignment of bisulfite-treated reads to the reference genome is evidently an important step during downstream processing. Standard mapping tools are not suitable for these data due to the high number of converted bases which present as errors. Reduction of reporting error thresholds lead to a high proportion of false positive alignments, so specific tools have instead been developed to explicitly enable read mapping of bisulfite data. Choosing the right tool can be daunting for scientists without formal training in bioinformatics, and is influenced considerably by the context and scope of each study. Previous independent comparisons among such tools have focused on algorithmic differences [11], combinations of pre- and post-processing techniques [12] or a small range of tools on model data (e.g. human) [13, 14]. Such reviews help to refine computational best-practices during software development, but it is important also to consider the biological implications of emerging end-use cases such as those presented by non-model plant data.

Plant genomes are notoriously difficult to work with due to large, repetitive sequences, regions of low complexity and a variably high degree of ploidy and zygosity. These factors can confound both genome assembly and alignment, often resulting in low-quality genomes with poor contiguity and multiple misassemblies. With non-model species there is a greater likelihood that the genome will exist in a draft state. These issues are usually mitigated for example with long-read sequencing technologies, such as PacBio or Oxford Nanopore, but fragmentation caused by the harsh sodium bisulfite treatment reduces the viability of such approaches during the present application.

In this study, a selection of nine, current, bisulfite short-read alignment tools are compared using a combination of real and simulated sequencing data, for three non-model plant species which vary in terms of genome composition and assembly quality (Table 1). These species are represented in the broader initiative of the EpiDiverse consortium^1^ , and include a high-quality (almost chromosome-level) assembly of the perennial Rosaceae *Fragaria vesca* [15] and two fragmented scaffold-level assemblies; one with higher repeat content in the case of the annual Brassicaceae *Thlaspi arvense* [16], and one with lower in the case of the unpublished, *de novo* assembly of the deciduous tree species *Populus nigra* (unpublished). Each species serves as a representative use case for other non-model organisms. The software are chosen in-part based on availability through Bioconda [17] (for reproducibility) and include *Bismark* [18], *BS-Seeker2* [19], *BSMAP* [20], *BWA-meth* [21], *ERNE-BS5* [22], *GEM3* [23], *GSNAP* [24], *Last* [25] and *segemehl* [26].

**Table 1:** Basic assembly statistics (approx.) for non-model plant species referenced in this study

Species	Genome size (Mb)	Scaffolds	Scaffold N50 (Mb)	Repeat content (%)	Accession	Source
*F.vesca*	220	29	33.9	33	Fragaria_vesca_v4.0.a1	rosaceae.org [31]
*T.arvense*	343	6,768	0.14	55	GCA_000956625.1	NCBI [32]
*P.nigra*	417	9,533	9.49	32	*unpublished*	*unpublished*

Note: Repeat content is given as a percentage of the total genome space.

Read mapping for each tool is evaluated in terms of precision-recall of the bisulfite-treated reads when compared to unique alignments of a corresponding, unconverted dataset mapped using the fully sensitive aligner RazerS 3 [27]. Futhermore, methylation profiles are derived from real data and the tools evaluated based on the mean absolute deviation of methylation values, using a subset of difficult-to-map regions where a $\log _2(x)>1$ absolute deviation in sequencing depth is observed overlapping a repeat annotation in at least one tool. Processing time and peak memory consumption are also measured over incremental levels of sequencing depth to assess the comparative performance of each tool on a standard computing architecture.

## 2 Materials and Methods

### Reference species

All species are non-model plant organisms selected under the broader initiative of the EpiDiverse consortium. Each reference varies in its overall assembly contiguity and underlying feature complexity (Table 1), representing different stages of assembly completeness. Repeat annotations were derived using EDTA [28].

### Natural accessions

To contrast features common to artificial reads and to infer the effect of read mapping on methylation quantification, one natural accession per species (150 bp long paired-end reads, randomly down-sampled to 20x) was mapped in addition to the simulated data. Methylation profiles were derived for each species by aggregating the methylation calls obtained following read alignment with each tested software. These profiles represent the underlying truth sets for then simulating artificial reads based on naturally occurring methylation patterns. A schematic describing the interaction between different datasets can be found in Supplementary Figure S1.

### Read simulation

Five independent sets of 125 bp paired-end reads were generated artificially from each reference genome using the read simulator Sherman v1.7 [29]. The datasets range incrementally from 1 to 20x sequencing coverage and were generated initially with a variable insert size ranging from 0 to 500, a random nucleotide error rate of 0.5% and a bisulfite conversion rate of 0. A variable length adaptor sequence was also generated, which was subsequently trimmed using cutadapt v2.5 [30]. The unconverted reads were then processed by an in-house script which applied a random 99% bisulfite conversion rate, yielding in the end two corresponding sets of simulated reads in FASTQ format, with and without bisulfite conversion. An additional set of artificial reads were converted from the 20x dataset in each species, using position-weighted conversion probabilities derived from the aggregate methylome obtained from the natural accessions.

### Read alignment

A total of nine current short-read mapping tools were selected to give a representation of current tools with different alignment strategies (discussed in more detail by Tran et al. [11]), with consideration given only to those with availability through Bioconda in the interest of reproducibility (Table 2). Each software was installed on a small server architecture housing 64 cpus with a total of 256 Gb memory (Supplementary Table S1). For testing purposes the tools were run with default parameters, which can be interpreted as the best approximation of a ‘general use case’. Relative processing time (real) and peak memory allocation (resident set size) are reported for each tool, utilizing a maximum of eight parallel threads so that results can be relevant to those working e.g. on a laptop or similar. Paired-end data from natural accessions were mapped both in paired-end and single-end mode, after obtaining the reverse complement of read 2 *in silico*, for comparison of mapping rates.

**Table 2:** Short-read alignment software tested in this study for mapping bisulfite sequencing reads. Equal-scoring alignments of multi-mapping reads are randomly selected as primary alignments where indicated, and otherwise not reported at all under default parameters

Mapping Software	Version	Default Reporting	Alignment Strategy	Index Structure
Bismark	0.22.3	unique best	3 letter	BWT (bowtie2)
BS-Seeker2	2.1.7	unique best	3 letter	BWT (bowtie2)
Last	1021	unique best	wild card	Spaced suffix array
BSMAP	2.90	unique best / random	wild card	Hash table (SOAP)
BWA-meth	0.2.2	unique best / random	3 letter	BWT (BWA)
ERNE-BS5	2.1.1	unique best / random	wild card	Hash table
GEM3	3.6.1	All-first-N / random	3 letter	Custom FM-index
GSNAP	2019-09-12	All-first-N / random	wild card	Hash table
segemehl	0.3.4	All / random	wild card	Enhanced suffix array

Note: BS-Seeker3 is available but was unable to run successfully on the provided computing infrastructure and has no recipe in Bioconda at the time of publication.

**Figure 1:**
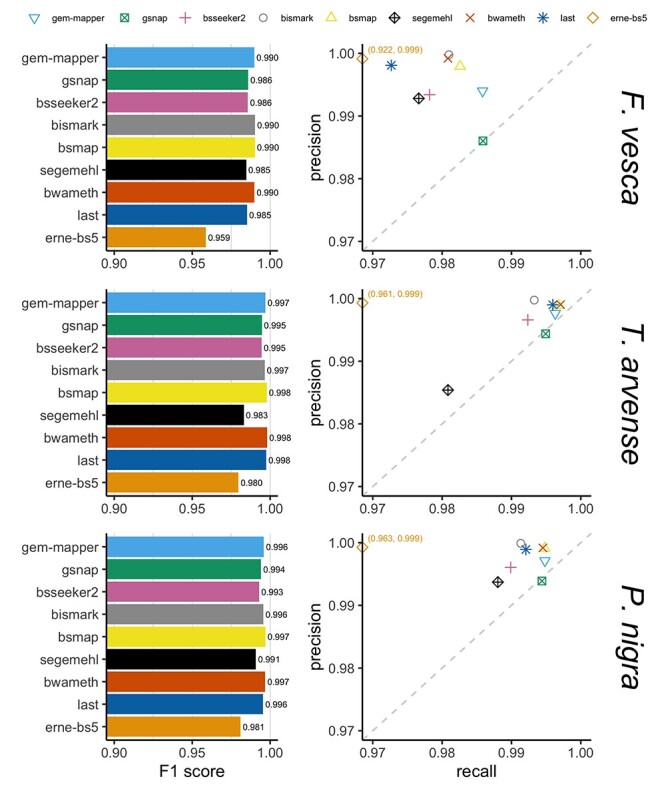
F1 scores and precision-recall for simulated reads mapped by each aligner, as determined by the equivalent alignment of unconverted reads by RazerS 3, demonstrating the response trade-off at close to maximum recall with a minimum mapping quality (MAPQ) threshold of 1. *BS-Seeker2* and *BSMAP* do not make use of MAPQ scores, and *ERNE-BS5* partitions alignments either at MAPQ = 0 or MAPQ = 60. The F1 score is the harmonic mean of precision and recall, which reflects the ranking of each tool relative to the overall balance of both measures. In the right-hand panels, ERNE-BS5 in each case falls out-of-bounds and is annotated with the appropriate coordinate (recall, precision).

**Figure 2:**
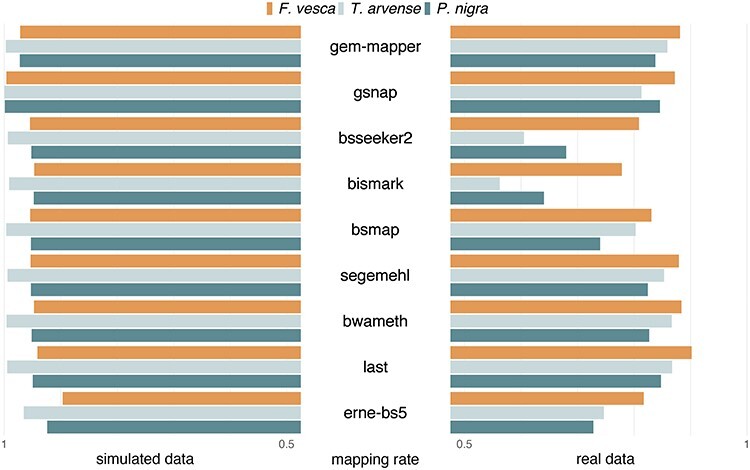
Mapping rates of short-read aligners. Comparisons between simulated and natural accession (real) data for each test species and each tool, given a minimum mapping quality (MAPQ) of 1. Reads from simulated data are generated from each corresponding reference genome and thus expected to behave concordantly, with little sequence variation and minimal influence of base quality, whereas real data may be subject to discordant alignments arising from poor reference contiguity and/or genomic rearrangement.

### Mapping rates

Read alignments from each tool were compared in both simulated data and natural accessions (real) data for each species in terms of the overall mapping rate for primary alignments with a minimum mapping quality (MAPQ) threshold of 1. On real sequencing data from natural accessions, mapping rates were calculated additionally for alignments of paired-end data in single-end mode, and also stratified by alignment edit distance (i.e. number of non-bisulfite mismatches) for paired-end alignments. Custom in-house scripting was used to obtain the appropriate edit distance where it was not reported by default by the alignment software.

### Precision-recall

Read alignments from each tool were compared to the point of origin of the read according to the metadata obtained from the read simulation tool. An additional truth set was also generated by aligning the unconverted reads to the reference with the fully sensitive aligner RazerS 3, discarding reads that aligned to multiple loci. The higher base complexity in unconverted reads gives an advantage to aligners compared with bisulfite-converted reads. The comparison between the truth set and the bisulfite read alignments allow for the identification of true positives, which demonstrate indirectly the false positives and false negatives derived by each method through the calculation of recall and precision (Supplementary Table S2). True positive alignments must occur in the same orientation and with the start coordinate within 5 bp of the corresponding alignment in the truth set. To limit the effect of sampling, the arithmetic means of precision and recall were calculated over all independent simulated datasets (1–20x) for each tool. Tools were then assigned an F1 score, which reflects the balance of precision and recall through calculation of the harmonic mean of both measures.

### Coverage deviation

Regions of $\log _2$-fold differential sequencing depth were calculated for each tool in comparison to unique RazerS 3 alignments using deepTools v3.4.3 bamCompare [33], after filtering bisulfite alignments based on a minimum MAPQ threshold of 1. The representation of such regions in the genome space of repeat annotations is analysed with a Fisher test implemented by bedtools v2.27.1 fisher [34]. Regions with a minimum absolute deviation in sequencing depth of $\log _2(x)>1$ in at least one tool are intersected with repeat annotations using bedtools v2.27.1 intersect [34], to identify a difficult-to-map subset of the genome space for comparative DNA methylation analysis.

### DNA methylation analysis

Methylation profiles for both natural accession data and artificial data were derived in all methylation contexts (i.e. CG, CHG, CHH) using MethylDackel v0.5.0 [35]. The tool adjusts for overlapping paired-end reads, and can account for methylation bias at the 5-end arising during library preparation due to unconverted nucleotides incorporated by end-repair. All alignments were filtered based on a minimum MAPQ score of 1, and positions with a minimum base quality of 1. The methylation calls from natural accession data, produced following alignment with each of the tested software, were combined into an aggregate methylome for use during read simulation of artificial data to confer position-weighted conversion probabilities from naturally occurring 5mC patterns. Resulting methylation calls from the simulated data, produced after aligning with each of the tested software, were then compared back the aggregate methylation profile over the difficult-to-map regions to evaluate the methylation differences in terms of mean absolute deviation.

**Figure 3:**
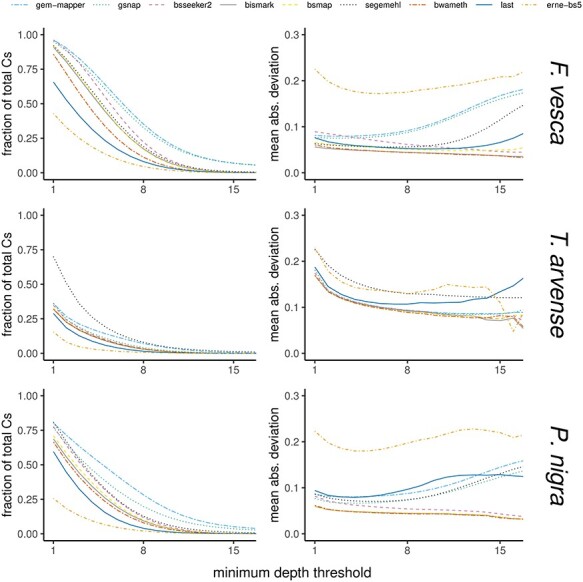
Fraction of total cytosines and mean absolute deviation of methylation calls. Comparisons between tested software in terms of the methylation profiles derived from simulated data, in all methylation contexts (i.e. CG, CHG and CHH), over difficult-to-map regions which encompass $\sim $3.75% of the genome space in *F. vesca*, $\sim $0.3% in *T. arvense* and $\sim $2.11% in *P. nigra*. All plots refer to profiles derived from artificial data simulated based on naturally occurring methylation patterns from the corresponding natural accession data. The left-hand panels show the fraction of total cytosines in difficult-to-map regions that are covered by each tool. The right-hand panels show the mean absolute deviation, demonstrating how well the methylation patterns were preserved following alignment with each tested software in comparison to the original methylation profiles from natural accession data.

## 3 Results

Precision-recall profiles derived from simulated read alignments demonstrate higher F1 scores when comparing to equivalent, uncoverted alignments obtained from RazerS 3 (Figure 1), but follow a similar behaviour in terms of dataset difficulty when comparing to the biological point of origin (Supplementary Figure S2), suggesting that the underlying feature complexity of each genome tested does not deter mapping beyond what can be expected from standard Illumina paired-end sequencing data. When filtering alignments by a minimum MAPQ threshold of 1, the aligners *BSMAP* and *BWA-meth* consistently exhibit the highest F1 scores across all datasets, followed closely by *Bismark*, *GEM3* and *Last*.

Despite a relatively high repeat content relative to the genome space and a highly fragmented assembly, *T. arvense* perhaps represents the most straightforward simulated dataset in this benchmark, since artificial reads originate only from within scaffolds so they have fewer potential loci to map back to. Conversely, *F. vesca* appears to be the most difficult despite its completeness and relative size. Comparisons with real data demonstrate lower mapping rates overall (Figure 2), particularly in less contiguous and less polished assemblies, possibly due in-part to the presence of discordant reads overlapping break points between scaffolds. *Bismark* and *BS-Seeker2* appear to be particularly susceptible to this, which can be unveiled by aligning the data in single-end mode (Supplementary Figure S3). The remaining gap can be largely explained by the fact that neither tool seems to output read alignments with more than four to five errors relative to other tools (Supplementary Figure S4). Taken together it results in fewer methylation calls for both of them (Supplementary Figure S5), which could potentially confound downstream methylation analysis.

As the difficulty of each dataset increases each tool tends to maintain a level of precision at the expense of recall, whereas *GSNAP* seems to traverse along the vector of y = x, and *segemehl* appears to struggle initially with the *T. arvense* dataset perhaps in-part due to the highly fragmented nature of the reference. The aligners *GEM3* and *BSMAP* tended to be among the most sensitive, except for the *F. vesca* dataset where *GSNAP* also recovered a greater proportion of positive alignments. The lowest recall was observed consistently for *ERNE-BS5*, which appears to apply a non-standard usage of MAPQ by binning alignments either at MAPQ = 0 or MAPQ = 60. This is reflected by a comparatively high precision relative to the other tools, similar to *Bismark* and *BWA-meth*. Further refinement of alignments in other tools by filtering MAPQ thresholds would likely result in improved levels of precision at the cost of recall, with the exception of *BSMAP* which does not make use of MAPQ. Given a minimum MAPQ threshold of 1, the aligners *segemehl* and *GSNAP* scored lowest in terms of overall precision.

Regions with an absolute deviation of sequencing depth of $\log _2(x)>1$ in at least one tool represent a total of $\sim $9.7 Mbp, $\sim $1.2 Mbp and $\sim $16.4 Mbp of the total genome space (4.39%, 0.34% and 3.92%), respectively, in *F. vesca*, *T. arvense* and *P. nigra*, whereas repeat annotations derived from EDTA comprise $\sim $73.4 Mbp, $\sim $190.1 Mbp and $\sim $135.2 Mbp. Independent F-tests of the intersection overlaps for each species indicate they are overrepresented in the genome space (${P}<1.0$x$10^{-6}$) at $\sim $8.3 Mbp, $\sim $1.0 Mbp and $\sim $16.4 Mbp (3.75%, 0.30% and 2.11%). These regions can be considered difficult-to-map, and the difference relative to RazerS 3 between the alignment tools is reflective of how multi-mapping reads are handled in relation to MAPQ (Figure 3).

In all cases it is expected that mean absolute deviation is inversely correlated with sequencing depth, as a greater number of overlapping reads should reduce the impact of spurious alignments. For some tools however the absolute deviation increases again for higher values of minimum sequencing depth in difficult-to-map regions, particularly in the range of >10x where the per-strand depth is greater than the expected mean (Figure 3). This indicates a tendency to map reads which likely differ in their point of origin, which is apparent to some extent in all software with ‘All’ or ‘All-First-N’ reporting strategies for multi-mapping reads, and additionally *ERNE-BS5* (random best) and *Last* (unique only). The influence of such alignments from these tools may be curtailed by setting upper limits for sequencing depth or by more stringent filtering on MAPQ.

Comparisons of the mean deviation in methylation rate over all positions as a function of a threshold on the minimum sequencing depth within difficult-to-map regions indicate that all software with the exception of *ERNE-BS5* differ only marginally from the expected methylation rate in natural accessions (Supplementary Figure S6), at lower depth thresholds, regardless of the recovered fraction of independent sites that are called (Figure 3). A higher rate indicates a potential preference towards aligning methylated reads, which could have implications for downstream methylation analysis in such regions. The tendency is not apparent when considering the global methylation profile across the whole genome.

**Figure 4:**
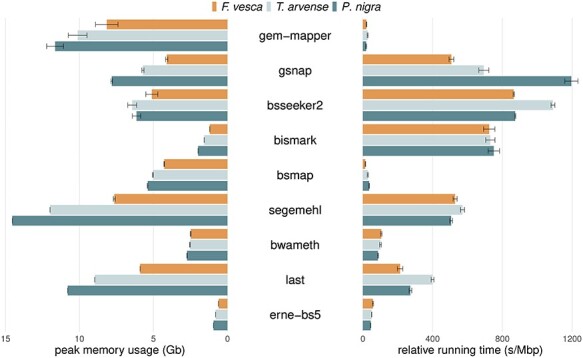
Peak memory and running time on alignments of simulated reads at varying levels of sequencing coverage (1–20x). Peak memory usage is given in terms of resident set size (Gb) and running time in terms of seconds per Mbp for comparison. Memory is dependant on the size of the genome relative to the effect on the index data structure, whereas time is dependant on the total quantity of reads to align. Larger error bars indicate memory usage differences that arise due to differences in sequencing depth, or non-linear increases in process running time.

The aligners *BSMAP*, *BWA-meth*, *ERNE-BS5* and *GEM3* exhibited the fastest running times, while *BWA-meth* and *ERNE-BS5* also ran with the lowest demand on peak memory alongside *Bismark* (Figure 4). For production environments with a focus on high throughput, aligners such as *BWA-meth* and *ERNE-BS5* might be preferred. If computational resources are not a factor then on-balance *BWA-meth* and *BSMAP* are able to make the most of the data available, depending on whether further refinement by MAPQ is required. For non-model data specifically, further consideration might also be given to how discordant alignments are handled by each tool.

## 4 Discussion

Previous studies have shown the imperative to consider methodological differences in the context of downstream methylation analysis, for example when detecting bias in WGBS library preparation strategies [36]. When mapping bisulfite-converted short reads, prioritizing one of either recall or precision might be appropriate when assessing individual alignments but can lead to bias in methylation rates. Deriving the correct result over a given position is dependant on maintaining the correct ratio of methylated and unmethylated cytosines from the pool of reads obtained from the biological sample. This ratio is disturbed not only by inaccurate mapping, as can be more prevalent in software with lower precision, but also by over-filtering alignments based on measures such as MAPQ, as may be prevalent in software with lower recall. The trade-off is more apparent when considering the stringency for handling multi-mapping reads in each tool with respect to MAPQ, particularly over difficult-to-map regions with local minima or maxima in overall sequencing depth.

Adjusting methylation rates or providing confidence intervals based on the evaluated mappability of reference regions might be beneficial for downstream analysis; however, existing tools based on self-alignments of k-mers may overestimate the mappability of heterozygous loci and/or scaffold boundaries in highly fragmented genomes [37]. Furthermore, differences in mean methylation patterns between different software indicate preferences in some instances for mapping methylated loci which are not explained by sequencing depth bias arising through library preparation. More densely methylated reads benefit from increased sequence complexity, which may confer an advantage during read alignment which has a downstream impact on methylation rate. The performance of WGBS alignment software is responsive to achieving an optimal balance of precision-recall with respect to both methylation status and the mappability of genomic regions.

It is important to consider that the metrics typically used in benchmarking approaches tend to reflect only the descriptive statistics of individual cases; they do not account for the full breadth of potential variation between different species. Though model species are often used to make predictions, a more robust statistical approach would strictly be necessary in order to develop a high-confidence model that carries over to other, non-model organisms. In the present context, the benchmarking of software using their default parameters appears most fair as an approximation of a ‘general use case’ and also trivial for any educated user to carry over to other scenarios. Parameter optimization is dependent on consistent implementation and reproducible behaviour between use cases, and we do not expect an educated user to select optimal settings for each tool without assistance by an expert. In summary, this study expands upon existing work by incorporating a range of emerging applications and shifting focus towards downstream methylation analysis; however, further refinement is encouraged on a case-by-case basis both in terms of software selection and the optimization of parameter settings to further improve results.

**Key Points**

- Precision-recall analysis of nine tools for mapping whole genome bisulfite sequencing data reveals on-balance that BWA-meth and BSMAP achieve consistently high F1 scores across all three non-model plant datasets. These tools were also among the best-performing in terms of peak memory consumption and running time.
- It is important to consider the balance of both precision and recall as they each have a direct influence on downstream methylation analysis.
- Particularly in regions of poor mappability, the handling of multi-mapping reads with respect to mapping quality (MAPQ) scores and the increased sequence complexity of densely methylated reads can potentially lead to bias in downstream methylation results.
- In non-model organisms with fragmented or less-polished genomes, the stringency of internal software constraints on mate pairs and allowed number of mismatches can explain differences in mapping rates between real and simulated data.

## Supplementary Material

supplement_bbab021

## Data Availability

Simulated reads, alignments and methylation bedGraphs are available on reasonable request and otherwise hosted at https://epi.bioinf.uni-leipzig.de/benchmarking/. The code to reproduce results and figures is available at https://github.com/bio15anu/benchmarking/. Third-party data, including the unpublished *P. nigra* genome and raw reads from natural accessions, will be made publicly available post-publication but are otherwise available on reasonable request and with permission from the EpiDiverse consortium.

## Footnotes

1
https://www.epidiverse.eu/

## Notes

**Adam Nunn** is a PhD candidate in Bioinformatics at the University of Leipzig, and is based at ecSeq Bioinformatics GmbH. His research focuses on the application of NGS analyses to bisulfite-sequencing data and the development of best-practice pipelines for studying non-model plant species.

**Christian Otto** received his doctorate in Bioinformatics from the University of Leipzig in 2015. He is one of the developers of the NGS read aligner segemehl and has also developed software tools to study the methylome by analysing bisulfite-treated sequencing data.

**Peter F. Stadler** received his PhD in Chemistry from U. Vienna in 1990 and then worked as Assistant and Associate Professor for Theoretical Chemistry at the same school. In 2002 he moved to Leipzig as Full Professor for Bioinformatics. He is External Professor at the Santa Fe Institute, External Scientific Member of the Max Planck Society, Corresponding Member Abroad of the Austrian Academy of Sciences, and Profesor honorario at the Universidad Nacional de Colombia.

**David Langenberger** received his doctorate in Bioinformatics from the University of Leipzig in 2013. He is now chief executive officer and co-founder of ecSeq Bioinformatics GmbH, and his research focuses on analysing NGS data and designing software tools to simplify these analysing processes.
