# Erratum to: Comprehensive benchmarking of software for mapping whole genome bisulfite data: from read alignment to DNA methylation analysis

**DOI:** 10.1093/bib/bbab183

**Published:** 2021-04-22

**Authors:** Adam Nunn, Christian Otto, Peter F Stadler, David Langenberger

**Affiliations:** ecSeq Bioinformatics GmbH, Sternwartenstraße 29, 04103, Saxony, Germany; Institut für Informatik, Universität Leipzig, Härtelstraße 16-18, 04107, Saxony, Germany; ecSeq Bioinformatics GmbH, Sternwartenstraße 29, 04103, Saxony, Germany; Institut für Informatik, Universität Leipzig, Härtelstraße 16-18, 04107, Saxony, Germany; ecSeq Bioinformatics GmbH, Leipzig, Germany

In the originally published version of this manuscript, the corresponding author, and their affiliation, was designated incorrectly during the production process. The correct corresponding author of the paper is Dr David Langenberger.

This has now been corrected online and the Publisher apologizes for this error.

