# Supplementary material for: Comprehensive benchmarking of software for mapping whole genome bisulfite data: from read alignment to DNA methylation analysis
Source: Brief Bioinform. 2021 Feb 23;22(5):bbab021. doi: 10.1093/bib/bbab021 (PMC8425420; doi:10.1093/bib/bbab021)
Supplement: supplement_bbab021 [file supplement_bbab021.docx]

| **Supplementary Table S1.** Test system specifications. | |
| --- | --- |
| **Operating System** | CentOS Linux 7 (Core) |
| **Architecture** | x86_64 |
| **CPU Model** | Intel(R) Xeon(R) Gold 6130 |
| **Clock Speed** | 2.10 GHz |
| **Available CPUs** | 64 |
| **Available RAM** | 256 Gb |
| **File**  **System(s)** | xfs  ext4 |

| **Supplementary Table S2.** The confusion matrix indicating the relationship between true positives and false positives. Precision is calculated by taking the true positives as a proportion of the predicted condition positives, and recall is calculated by taking the true positives as a proportion of the true condition positives. The F1 score is the harmonic mean of precision and recall. All equations are denoted in the footnote. | | | |
| --- | --- | --- | --- |
|  |  | **True Condition** | |
|  |  | Positives | Negatives |
| **Predicted Condition** | Positives | True Positives | False Positives  (Type II error) |
|  | Negatives | False Negatives  (Type I error) | True Negatives |

$$precision= {True positives}/\left( True positives+False positives \right)$$

$$recall= {True positives}/\left( True positives+False negatives \right)$$

$$F1 score= 2\cdot\left( \left( recall\cdot precision \right)/\left( recall+precision \right) \right)$$

| **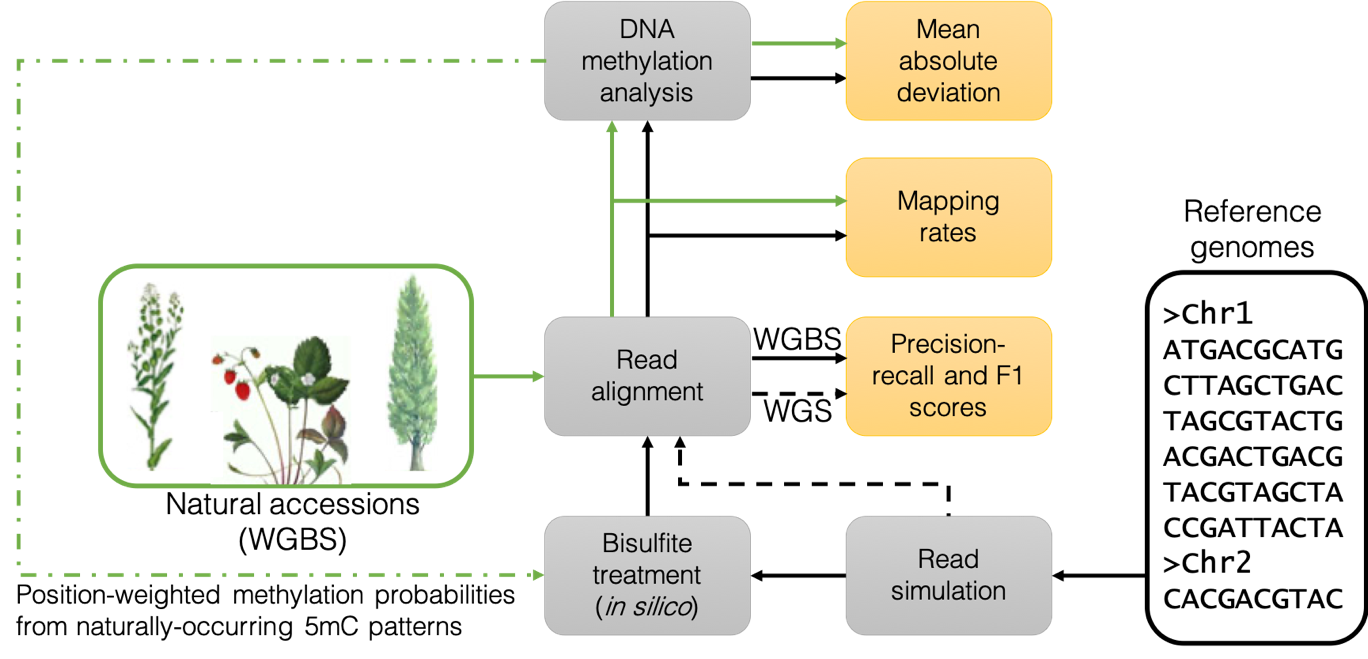** |
| --- |
| **Supplementary Figure S1.** Schematic overview of the experimental design used in this study. Note that grey boxes illustrate procedures whereas yellow boxes correspond to benchmarking measures. Also, processing of simulated and real data is indicated by black and green arrows, respectively. Naturally-occurring 5mC patterns derived from natural accessions are used to inform bisulfite treatment of simulated reads *in silico*. The simulated WGBS data is mapped with each tested aligner and compared to RazerS 3 alignments of untreated reads in order to evaluate precision-recall. The alignments are also compared to alignments from natural accessions by calculating mapping rates. Methylation profiles derived from simulated data are compared to naturally-occurring 5mC patterns in order to evaluate the influence of each software on downstream DNA methylation analysis. |

| 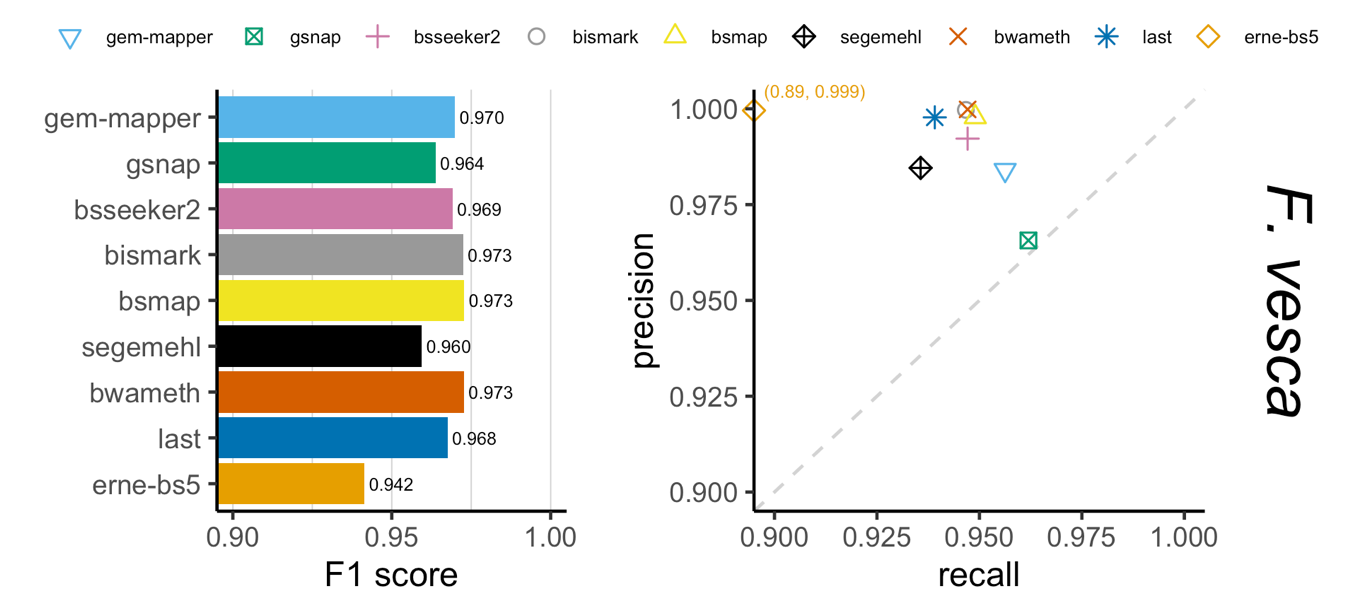 |
| --- |
| 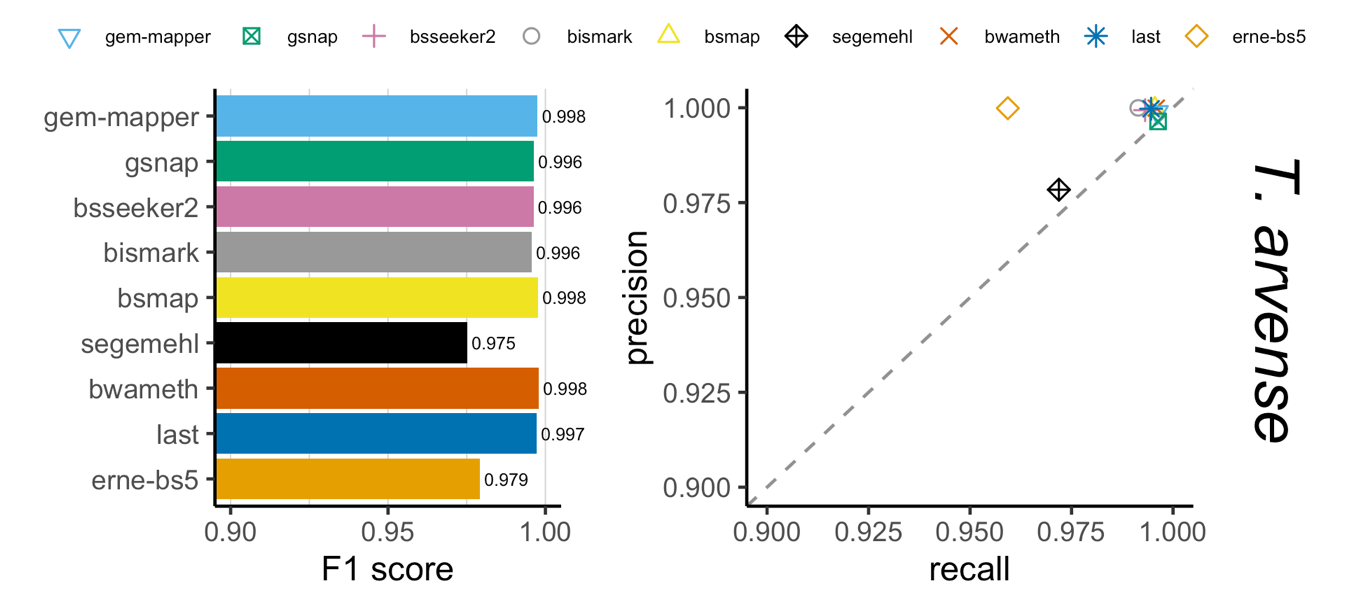 |
| 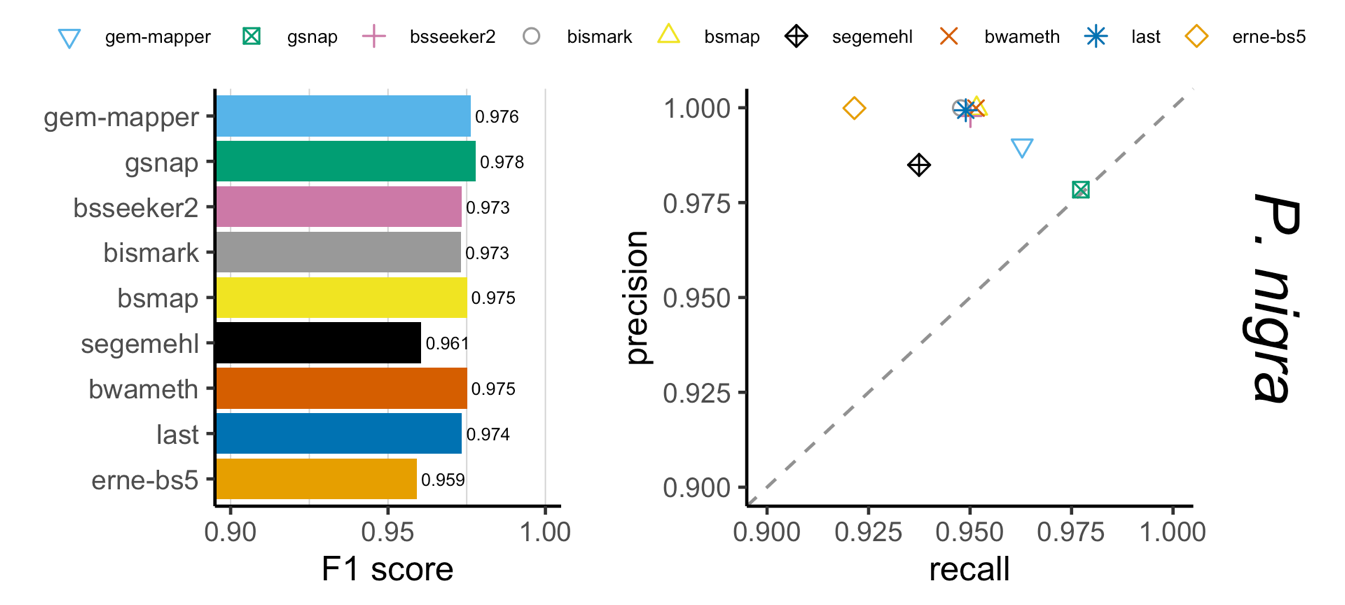 |
| **Supplementary Figure S2.** F1 scores and precision-recall for simulated reads mapped by each aligner, as determined by the known biological point of origin of reads according to the read simulator, demonstrating the response tradeoff at close to maximum recall with a minimum mapping quality (MAPQ) threshold of 1. BS-Seeker2 and BSMAP do not make use of MAPQ scores, and ERNE-BS5 partitions alignments either at MAPQ=0 or MAPQ=60. The F1 score is the harmonic mean of precision and recall, which reflects the ranking of each tool relative to the overall balance of both measures. In the right-hand panel for *F. vesca*, ERNE-BS5 falls out-of-bounds and is annotated with the appropriate coordinate (recall, precision). |

| **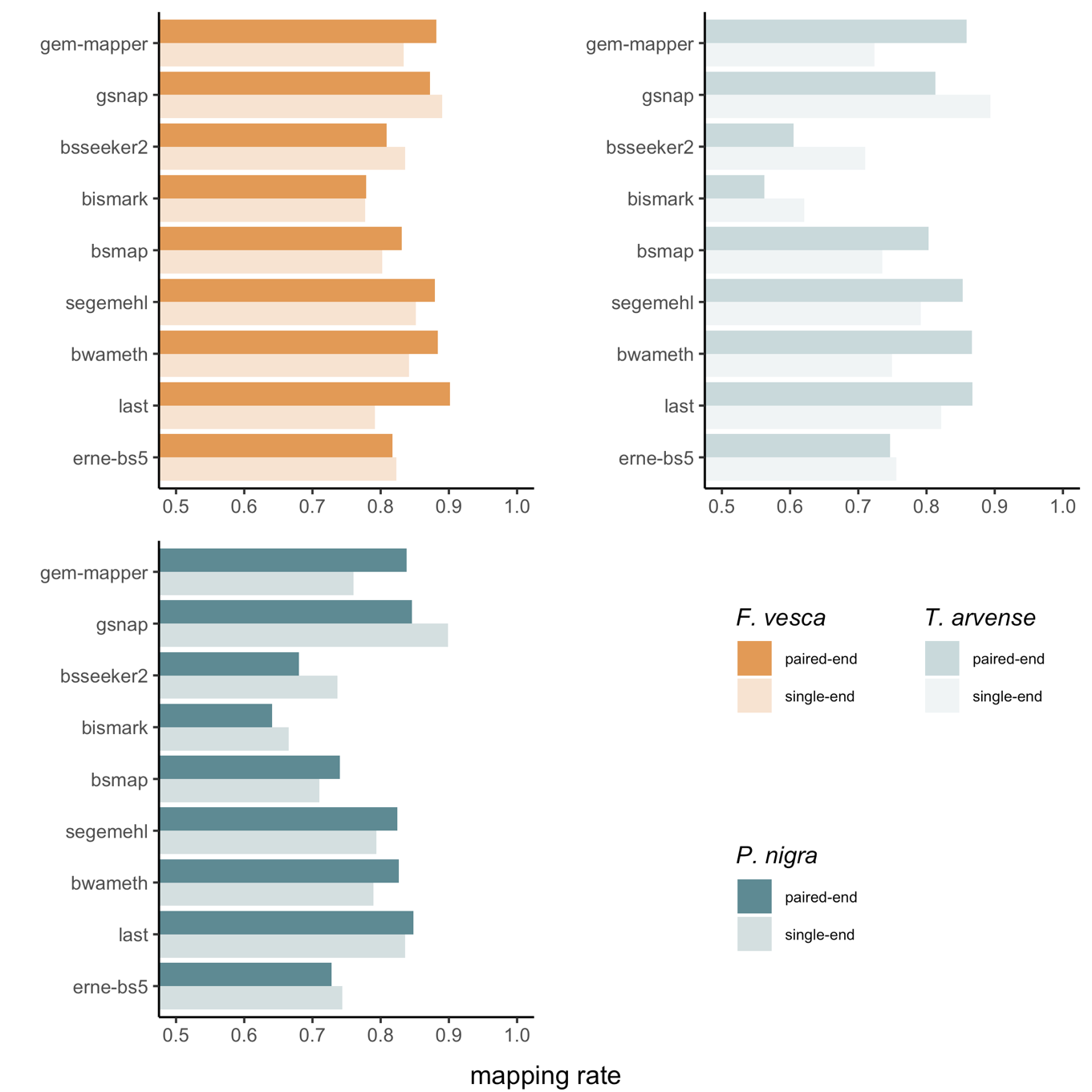** |
| --- |
| **Supplementary Figure S3.** Comparison of mapping rates with each software after aligning the same paired-end WGBS reads from natural accession (real) data in each species, in both paired-end mode and single-end mode. Most tools achieve marginally higher mapping rates in paired-end mode, with the exception of *Bismark*, *BS-Seeker2*, *ERNE-BS5* and *GSNAP*. In addition, both *Bismark* and *BS-Seeker2* appear to lose sensitivity in paired-end mode in correlation with an increasing level of fragmentation in the corresponding genome assemblies. |

| **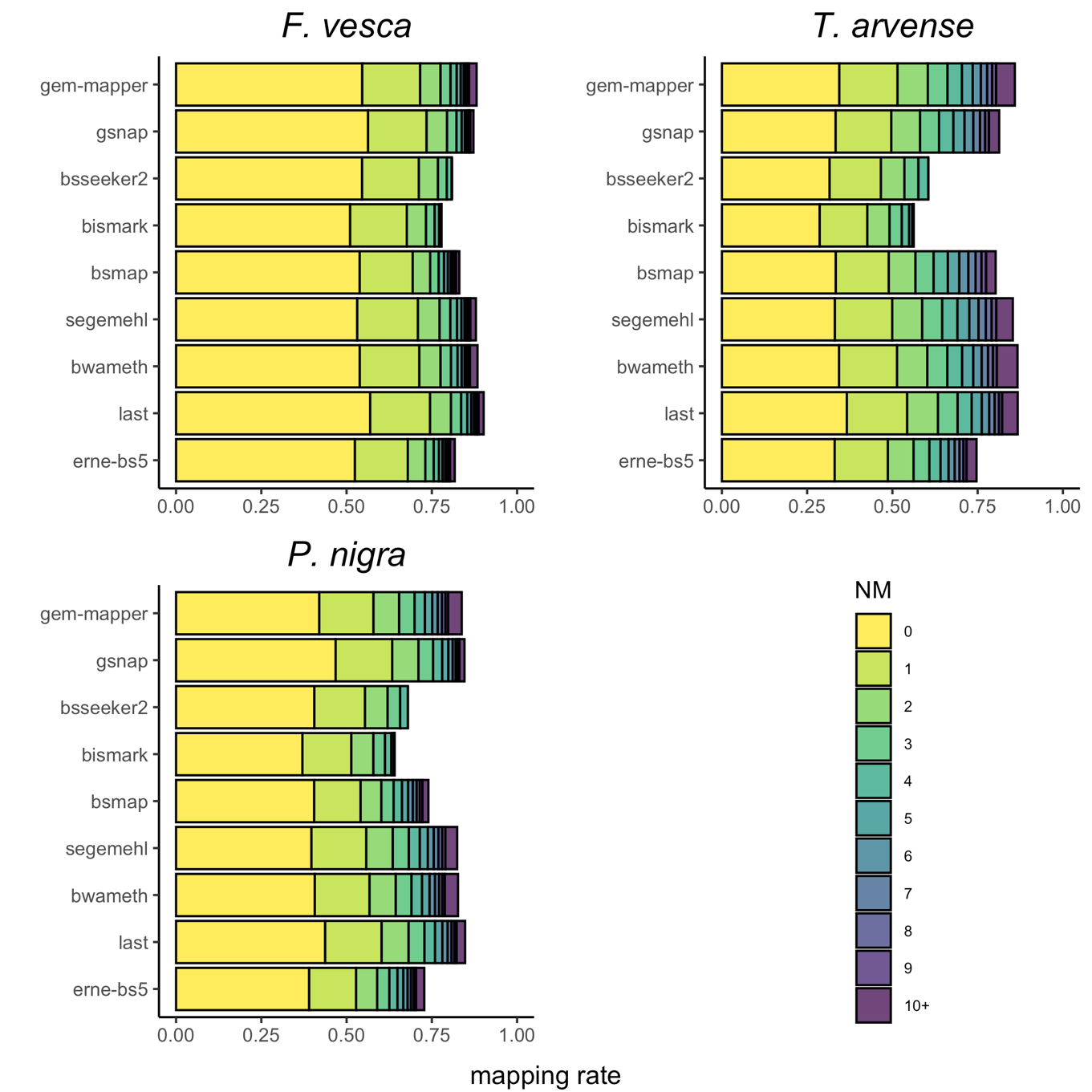** |
| --- |
| **Supplementary Figure S4.** Comparison of mapping rates with each software for paired-end alignments from natural accession (real) data in each species, stratified by the number of alignment errors (NM) which cannot be attributed to the treatment with bisulfite. Most of the aligners allow for up to ten or more errors in the read alignments and show only slight differences in the fraction of read alignments below a certain number of errors. The exceptions are *Bismark* and *BS-Seeker2* which appear to have a soft-/hard-threshold at 4-5 errors per read. |

| relative proportion of cytosines | 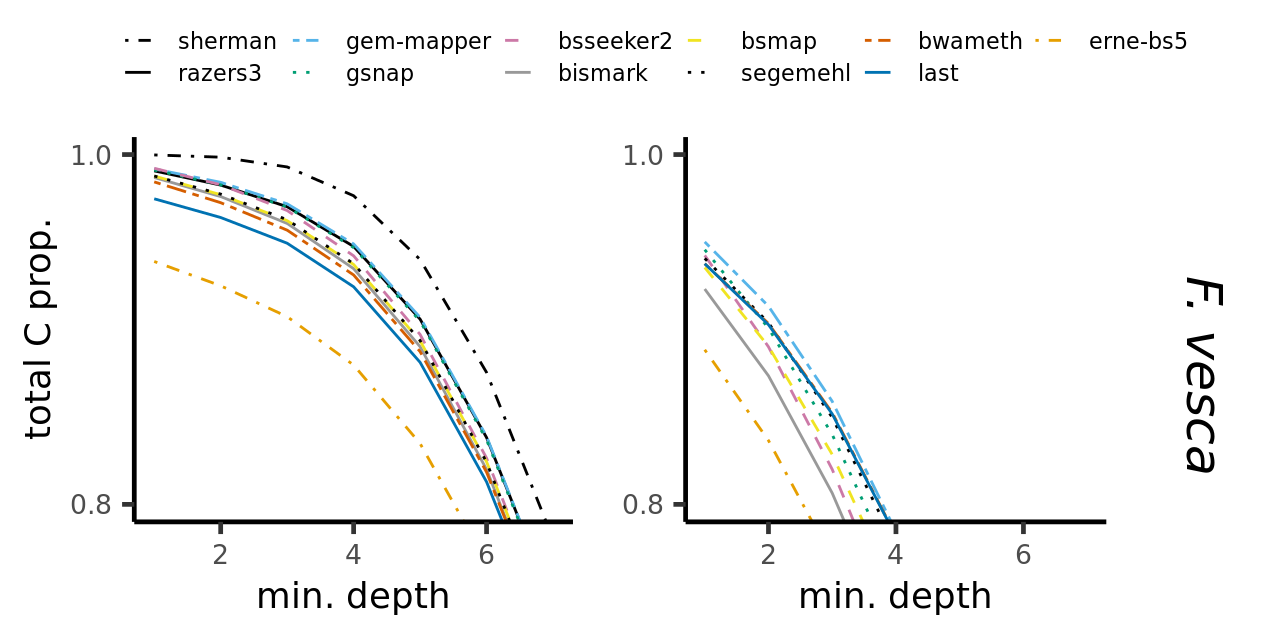 |
| --- | --- |
|  | 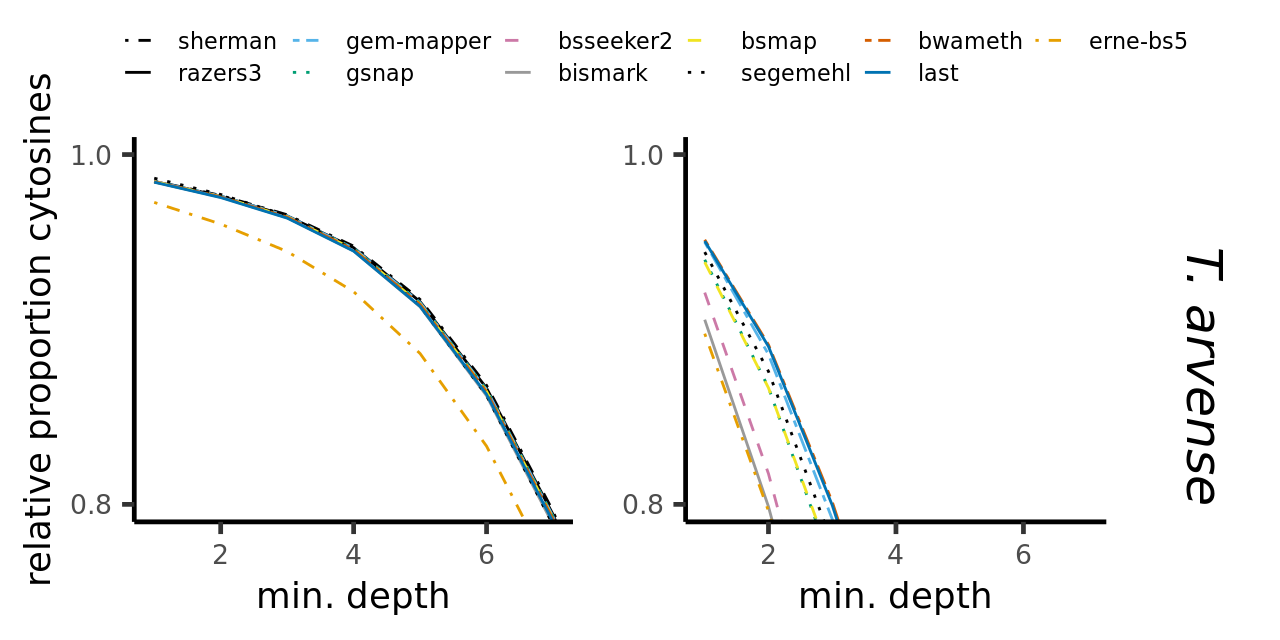 |
|  | 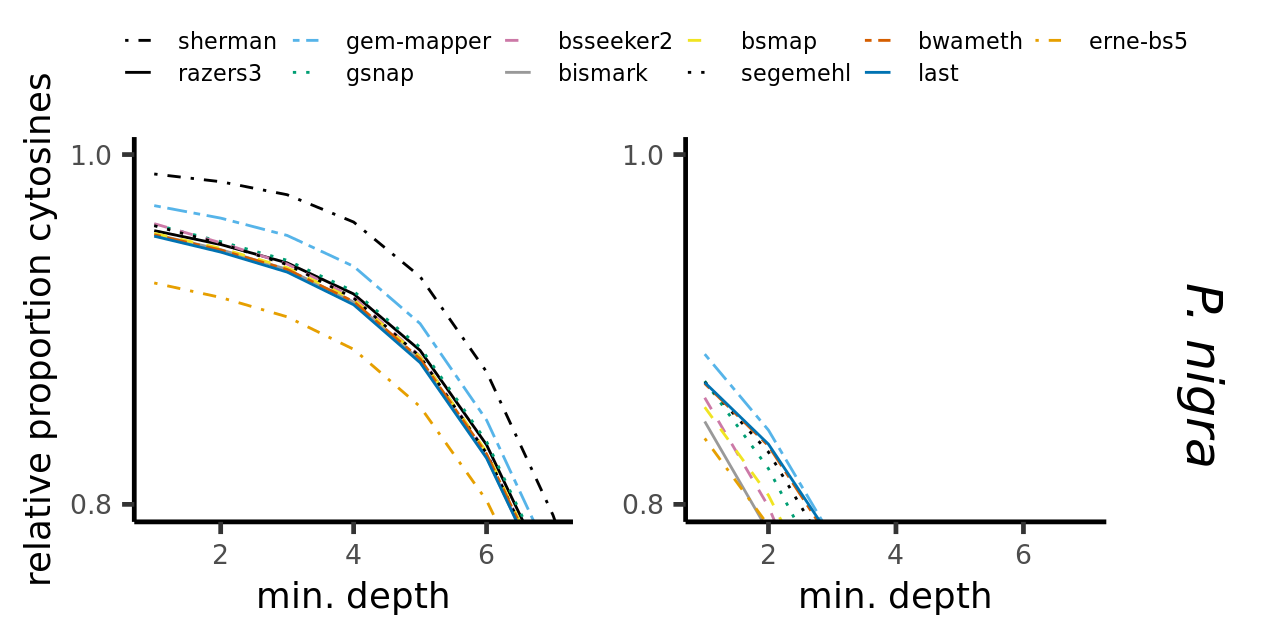 |
| **Supplementary Figure S5.** Global methylation site dropout. Total proportion of genomic cytosines in all methylation contexts (i.e. CG, CHG, CHH) derived from each aligner in response to varying the minimum sequencing depth threshold. The expected mean strand-specific sequencing depth is 10x. The left-hand panels represent simulated data, whereas the right-hand panels represent natural accession (real) data. | |

| **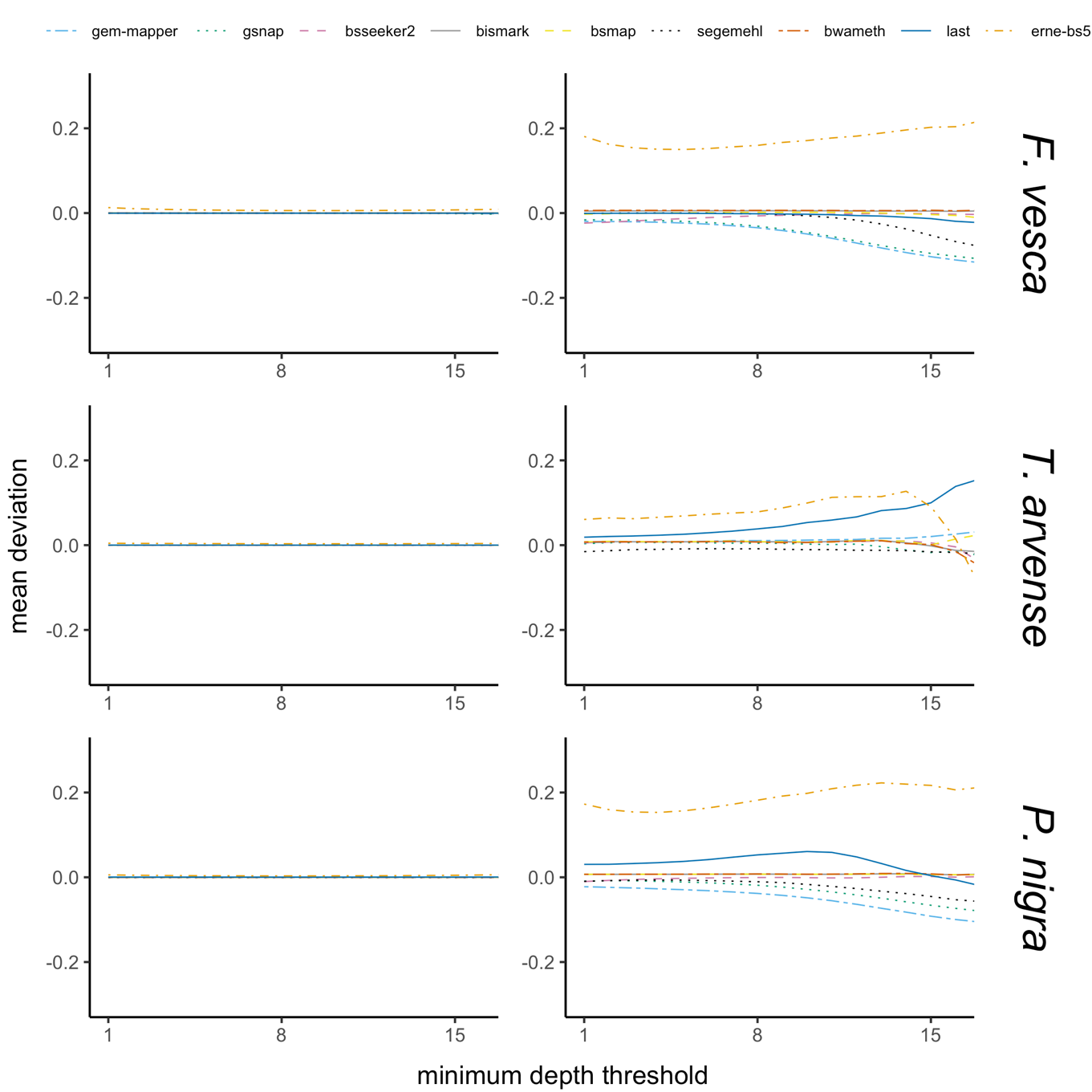** |
| --- |
| **Supplementary Figure S6.** Mean deviation of cytosine methylation (all contexts) in simulated data, relative to the naturally-occurring 5mC patterns derived from natural accessions, as a function of a threshold on the minimum sequencing depth after aligning to each species with each tool. The left-hand panels show the deviation on a genome-wide (global) scale whereas the right-hand panels show only the subset of difficult-to-map regions. Most software tools perform similarly on either scale, but few tend to underestimate the methylation level in difficult-to-map regions at higher-than-expected levels of strand-specific sequencing depth (e.g. >10x). In contrast, *ERNE-BS5* appears to overestimate the methylation level in difficult-to-map regions but otherwise does not differ noticeably from the global mean methylation level. |
